# Low awareness of venous thromboembolism among neurosurgical hospitalized patients: a cross-sectional study

**DOI:** 10.1186/s12959-023-00466-7

**Published:** 2023-03-16

**Authors:** Pan Lin, James Allen Wiley, Lingyun Tian, Wan Li, Qiuhong Yang, Haifan Yang, Xin Tan, Yijing Gao, Weijuan Li, Hui Luo, Xinyu Feng, Yinglan Li

**Affiliations:** 1 Xiang Ya Nursing School, Central South University (CSU), NO.172 Tongzipo Road Yuelu District, Changsha, 410013 Hunan Province China; 2 Department of Family and Community Medicine, University of California, San Francisco, USA; 3 Department of Nursing, The First Affiliated Hospital of University of Science and Technology of China (USTC), Division of Life Sciences and Medicine, University of Science and Technology of China, Hefei, 230001 Anhui China; 4 Cardiac Surgery Intensive Care Unit (CSICU), Xiangya Hospital of Central South University, No. 87, Xiangya Road, Kaifu District, Changsha City, 410008 Hunan Province China; 5 School of Nursing, Xinjiang Medical University, NO.393 Xinyi Road Xinshi District, Urumqi, 830000 Xinjiang Uygur Autonomous Region China

**Keywords:** Venous thromboembolism, Deep vein thrombosis, Pulmonary embolism, Prophylaxis, Neurosurgery hospitalized patients, Knowledge, Patient education and advocacy

## Abstract

**Background:**

Venous thromboembolism (VTE) including Deep Venous Thrombosis (DVT) and Pulmonary Embolism (PE), is a serious cause of patient morbidity and mortality in hospitals. Neurosurgical hospitalized patients have higher rates of immobility and bed rest, thus increasing their risk of developing VTE. This highlights the need for their thromboprophylaxis regimens. Patients’ awareness of VTE is essential for promoting strategies such as early ambulation and encouraging self-assessment and self-reporting of VTE signs and symptoms. This study evaluated neurosurgical hospitalized patients’ awareness of VTE and explored the influencing factors to provide a theoretical basis for nursing intervention.

**Methods:**

We selected one tertiary level hospital in Hunan Province and randomly sampled eligible patients from each five neurosurgical units. We conducted a cross-sectional survey of the hospitalized patients of neurosurgery using the self-designed and validated VTE knowledge questionnaire, and the influencing factors were analyzed using SPSS 26.0.

**Results:**

A total of 386 neurosurgical hospitalized patients completed the survey. The score of VTE knowledge in neurosurgical hospitalized patients was 13.22 (SD = 11.52). 36.0% and 21.2% of participants reported they had heard of DVT and PE, respectively. 38.9% of participants were unable to correctly identify any symptoms of VTE. The most frequently identified risk factor was ‘immobility or bed rest for more than three days’ (50.0% of participants), and 38.1% of patients agreed that PE could cause death. 29.5% of participants were unable to identify any prophylactic measures of VTE. The results of Negative Binomial Regression showed that the influencing factors of VTE knowledge in neurosurgical hospitalized patients were education level (*P* < 0.004) and sources of information related to VTE, including nurses (95% CI = 2.201–4.374, *P* < 0.001), and family member/friend (95% CI = 2.038–4.331, *P* < 0.001), Internet/TV (95% CI = 1.382–2.834, *P* < 0.001). Other sources included patient /pamphlet/poster /professional books (95% CI = 1.492–3.350, *P* < 0.001).

**Conclusions:**

This study demonstrates the lack of awareness of VTE among neurosurgical hospitalized patients. More attention must be paid to carrying out training on VTE knowledge according to different characteristics of neurosurgical hospitalized patients, so as to ensure safe and high-quality patient care.

## Introduction

Venous thromboembolism (VTE) refers to the abnormal clotting of blood in a vein that leads to venous return disorders, including deep venous thrombosis (DVT) and pulmonary embolism (PE). The occurrence of VTE is associated with considerable morbidity, mortality and costs. It is a constant risk in any hospital setting and a significant health issue on a global scale [1]. Studies have shown that the annual incidence of VTE in the adult populations is 1 in a 1000 [2], about 6% of DVT patients and 12% of PE patients die within 1 month of diagnosis of VTE [3]. According to a report, the annual economic burden of VTE in the United States is about $7 billion to $10 billion [4]. Thus, VTE brings serious disease burdens and economic burden to patients.

VTE is one of the major comorbidities in neurosurgical hospitalized patients [5]. Studies have shown that among hospitalized patients, neurosurgical patients have one of the highest risks of VTE, belonging to the high-risk group of VTE [6]. The American Society of Hematology 2019 guidelines for the management of VTE also noted that patients undergoing invasive neurosurgery had the highest risk of VTE among all surgical patients [7]. In China, the reported incidence of lower extremity DVT in postoperative neurosurgical patients was 31.1% [8]. DVT may lead to a PE. According to a Japanese study, the incidence of PE in neurosurgical patients with DVT was 60% [9]. An America study of neurosurgical patients revealed a PE risk of approximately 1.5%-5% with an associated mortality rate of approximately 9%-50%, close to 25% of all patients with PE will die suddenly [10]. Moreover, the clinical manifestations of VTE are often insidious and unpredictable, with few warning signs. Studies have shown that 60.7% of neurosurgical patients with lower extremity DVT were asymptomatic and only 39.3% of DVT were symptomatic [8]. However, doctors only diagnose VTE when patients show possible clinical symptoms, which often leads to missed diagnosis and misdiagnosis, and the actual incidence of VTE in neurosurgical hospitalized patients may be higher. Prophylaxis of VTE is, therefore, an integral part of neurosurgical patient care.

VTE can be prevented to a large extent [11]. Previous studies have shown that early preventive intervention can reduce the relative risk of DVT by 84% and PE by 55% in high-risk groups of VTE [12]. With the enhancement of patients' health and rights awareness, patients can actively obtain information related to disease prevention and treatment. In coordination with the implementation of diagnosis and treatment plan, participation in self-care management and rehabilitation exercise, self-reported outcomes play an increasingly important role in health management and health care. Similarly, improving the initiative of neurosurgical hospitalized patients to participate in the prophylaxis of VTE is of great significance for the promotion of patient safety. However, studies have shown that only on the basis of good disease knowledge can patients form correct disease prophylactic attitudes and promote healthy behaviors [13]. A previous survey conducted in the United States found that 74% of adults had poor knowledge of VTE and its complications [14]. If high-risk groups for VTE lack the correct knowledge of VTE, it is hard for them to realize their risk of VTE. Many patients are not actively participating in VTE prevention, which may cause the delay or failure of the clinical treatment, lead to the higher morbidity and higher mortality of VTE [15].

Although a number of general reports have focused on the importance of preventing VTE in neurosurgery [16], few studies have assessed neurosurgical hospitalized patients’ awareness of VTE. Therefore, in this cross-sectional study, we evaluated neurosurgical hospitalized patients’ awareness of VTE, and analyzed the factors affecting the knowledge level of VTE in neurosurgical hospitalized patients. This research helps nurses to formulate more scientific and effective VTE prevention and control programs and provides a theoretical basis for nursing intervention.

## Methods

### Ethical considerations

The study was approved by the Nursing and Behavioral Medicine Research Institutional Review Board, Xiangya Nursing School, Central South University (CSU) (IRB CSU: E202151). Written consent from the participants were obtained and confidentiality of their privacy was assured. The rights of participants to withdraw at any time were guaranteed.

### Design and sample

This was a cross-sectional study carried out from September to November 2021 at Xiangya Hospital, CSU, Changsha, China. The sample size was determined according to two calculation methods. First, sample size = variables × (5–10) × (1 + 20%) [17]. There were 11 items in the questionnaire, and 17 items in the general data questionnaire of neurosurgical hospitalized patients, with a calculated sample size of 168–336. Second: sample size was calculated according to the statistical formula: *n* = ${\left[\frac{\left({t}_{\alpha /2}+{t}_{\beta }\right)S}{\delta } \right]}^{2}$ [18], Where ‘n’ is the sample size, ‘S’ is the standard deviation and ‘δ’ is the allowable error. The standard deviation (S) of VTE prevention knowledge score of neurosurgery inpatients obtained through pilot study validation was 19.281, The allowable error (δ) is set at 0.18S, α = 0.05, β = 0.10, *t*_*0.05/2*_ = 1.960, *t*_*β*_ = *t*_*0.10*_ = 1.282. According to the formula, *n* = 324, and on this basis, 15% was added to prevent sample loss. The sample size was calculated to be 373. Combining the two calculation methods, the maximum 373 were selected as the sample size to be investigated. The study sample consisted randomly selected patients from five neurosurgery units (numbers of patients selected ranged from 57 to 99) for a total of 386 hospitalized patients who met the inclusion and exclusion criteria. There was no significant difference in VTE knowledge score among the five neurosurgical units (*P* = 0.375).

Surveys of self-reported experiences and retrospective medical chart reviews were conducted with the sample of patients. Participants of the study were hospitalized adults aged 19 years or older in the department of neurosurgery. The routine VTE prevention measures in the hospital included chemical prophylaxis in the form of low molecular weight heparin (LMWH), and warfarin and mechanical prophylaxis as recommended by the 2020 American Society of Hematology (ASH) VTE prevention clinical guidelines [2]. Patients on the 48-beds neurosurgical units were screened for inclusion criteria: 1) hospitalization in the department of neurosurgery, 2) age > 18 years, 3) written informed consent, voluntary participation in this investigation, and 4) no delirium or dementia as per medical chart. Patients were excluded from the study if they were 1) experiencing a lack of language proficiency, 2) with coma and disturbance of consciousness, and 3) receiving palliative care or being a terminal patient.

### Data collection

First, the corresponding author contacted the head nurse of the five neurosurgical units of Xiangya Hospital, CSU. Permission was also received from Xiangya Hospital, CSU to apply the data collection tools, an interview was chosen as a method to complete the questionnaires.

Before the investigation: All investigators were convened by the researcher under the supervision of the supervisor of the research group to conduct unified training. The training contents and methods included VTE prevention knowledge, the contents and methods of this study, the hospital medical record information system, precautions for questionnaire collection and nurse-patient communication skills, Possible errors and problem strain methods and so on. After the training they were assigned to different neurosurgical wards to perform the study.

Patients were screened for inclusion criteria and exclusion for current cognitive impairment based on data from the medical charts, nurses on the unit and assessment by the interviewers. Neurosurgical hospitalized patients were approached by the investigator and after declaring their interest to participate in the study, they received detailed information about the aim of the study and were asked to provide informed consent.

In the investigation process: all the investigators followed the principle of objective facts to conduct the investigation, and checked the integrity and authenticity of the questionnaires collected on site. Patients could ask the investigators if they did not understand anything in the filling process, and the investigators explained the problems without any induction or inspiration, then the patients completed the work by themselves. A small number of subjects may had poor eyesight, low educational level or difficulty in filling out their own information, the investigator would read the questionnaire items one by one and explained the questions, the patient then explained the specific choice of each item.

Four hundred and seventy-one neurosurgical hospitalized patients were contacted, but 85 (18.04%) refused to participate in the study. Most of the reasons for rejection were that the hospitalized patients could not see clearly or were busy going through discharge relevant procedures. The investigators administered the questionnaires in the privacy of the patients’ rooms. Survey administration was completed in approximately 10–20 min. The medical records of participants were reviewed by the investigators to identify patients' disease diagnosis and hospital stay. Diagnosis was based on discharge diagnosis, which was divided into 8 categories: craniocerebral injury, intracranial tumor, cerebrovascular disease, scalp and skull disease, intracranial infection, spinal cord disease, functional diseases, and others, respectively. The length of hospitalization was from most recent hospital admission date to the survey date. If the patient had been admitted to the neurosurgery department multiple times, the length of previous hospitalization was added. Participants each received a toothpaste compensation for their time and efforts to complete the questionnaire.

### Survey instruments

#### The process of questionnaire compilation

Initial draft of questionnaire: We did not identify any VTE preventive knowledge instruments specifically designed for neurosurgical hospitalized patients. The research group members drafted the original version questionnaire after referring to the questionnaires utilized in similar contexts such as application in orthopedic patients [18]. From June to August 2021, the Delphi method [19] was used to carry out expert consultation through correspondence, a panel of 11 Clinical nurses (come from neurosurgery, neurosurgical intensive care unit, vascular surgery, cardiac surgery), 4 Clinical doctors (including Neurosurgery and Hematology), 4 nursing administrators and 1 schoolteacher were invited to ask any question, make comments to any part of the questionnaire, after 2 rounds of correspondence, the first draft of the questionnaire contains 4 dimensions and 11 questions were formed. Ten hospitalized patients of neurosurgery were invited to conduct a small sample test with a pre-test questionnaire, and the content of the pre-test questionnaire was adjusted according to the feedback of patients, to ensure the intelligibility of the questionnaire.

Pilot study validation: Then the second draft of the questionnaire was pilot-tested with a sample of 210 neurosurgical hospitalized patients who met the cross-sectional inclusion and exclusion criteria. The time required to complete the questionnaires was approximately 5–10 min for each individual. The discriminant degree analysis method, correlation coefficient method and exploratory factor analysis (EFA) were used to conduct item reduction. All items’ critical ratio > 3.000 (*P* < 0.001), and the correlation coefficient between each item and the total score was ranged from 0.454–0.748 (*P* < 0.001). The Kaiser-Mayer-Olkin (KMO) value was 0.905 and Bartlett’s test was 1271.228, degrees of freedom was 55 (*P* < 0.001), which was adequate for EFA. Two factors were extracted by using rotated factor analysis. The two factors jointly accounted for 62.234% variance observed. The loading value of all items were > 0.400 (Table 1). Factor 1 consists of three items, which are mainly about the recognition of VTE, DVT and PE. They are named as basic knowledge of VTE. Factor 2 includes 8 items, including the causes, risk factors, clinical manifestations, and prevention knowledge of VTE, which is named as VTE professional knowledge. The questionnaire finally formed consists of two dimensions and 11 items.

**Table 1:** Rotated factor analysis of questionnaire for VTE knowledge

Items	Factors		Common degrees
	1	2	
A1.1	0.724		0.541
A1.2	0.919		0.789
A1.3	0.807		0.625
A4.1		0.780	0.468
A1.4		0.702	0.567
A1.5		0.836	0.720
A2.1		0.861	0.745
A2.2		0.865	0.671
A3.1		0.677	0.651
A4.2		0.746	0.668
A4.3		0.465	0.439
Eigenvalue	1.016	5.867	
Variance explained	9.240	53.335	

#### Content of the questionnaire

The survey consisted of two primary sections. The first section asked 17 questions concerning patient socio-demographic characteristics such as age, gender, area of residence, educational level, occupation, marital status, family monthly income (US dollars), payment manner of the medical expenses, the reason for admission, surgery, length of hospitalization, caregivers, sources of information related to VTE, whether there is a doctor/nurse in the family, personal history of VTE, family history of VTE, and whether the doctors/nurses have taught the patients about VTE knowledge. The second section consists of 11 questions measuring the knowledge of VTE (refer to the list in Table 2), including two sections: 1) The VTE basic knowledge, including three questions: ‘Have you heard of VTE?’, ‘Have you heard of DVT?’, and ‘Have you heard of PE?’ 2) The VTE professional knowledge, including nine questions: causes of VTE, the dangers of having VTE, symptoms and signs of DVT and PE, risk factors of VTE including ‘immobility or bed rest for more than three days, trauma, surgery, obesity, use dehydrating drugs, deep vein catheterization, have high blood pressure or diabetes and so on, infection, cancer/chemotherapy/radiotherapy, personal history of VTE’; preventive knowledge includes basic preventive knowledge, mechanical preventive knowledge, and drug preventive knowledge. For single choice, one point is given for "yes" and zero points is given for "no" or "uncertain". For sets of questions which could be marked, each choice except “not sure” is counted as one point (for example, “Which of the following are signs/symptoms of VTE: swelling, calf pain, redness and warmth of legs, lower extremity varicose veins, not sure). Finally, all the scores are added up to obtain the total knowledge score.

**Table 2:** Characteristics of Patients in neurosurgical hospitalized patients and univariate analysis (*n* = 386)

Factors	Patients N (%)	Knowledge score (`x±s)	*t/F*	*P*	Factors	Patients N (%)	Knowledge score (`x±s)	*t/F*	*P*
Age (years)			21.496#	0.001	Reason for Admission			0.297	0.955
Less than 25	15 (3.9)	14.20±9.80			Craniocerebral injury	17 (4.4)	12.12±14.15		
26-35	43 (11.1)	17.58±12.91			Intracranial tumor	232 (60.1)	13.41±11.10		
36-45	51 (13.2)	14.14±11.82			Cerebrovascular disease	72 (18.7)	13.88±12.21		
46-55	121 (31.3)	13.94±11.51			Diseases of the scalp and skull	5 (1.3)	9.00±15.26		
56-65	97 (25.1)	13.19±11.36			Intracranial infection	3 (0.8)	13.00±15.40		
More than 65	59 (15.3)	7.59±8.96			Spinal cord diseases	15 (3.9)	13.60±13.28		
Gender			0.348	0.728	Functional disease	35 (9.1)	11.40±10.70		
Male	186 (51.8)	13.42±11.47			Other	7 (1.8)	14.57±11.73		
Female	200 (48.2)	13.01±11.60			Surgery			3.194	0.002
Place of residence			5.441	0.000	Yes	195 (50.5)	15.05±12.46		
Rural areas	208 (53.9)	10.33±10.10			No	191 (49.5)	11.36±10.17		
Urban areas	178 (46.1)	16.60±12.18			Length of hospitalization			1.273	0.283
Education level			95.290#	0.000	Less than 7 days	272 (70.5)	12.55±11.15		
Primary and below	90 (23.2)	5.89±8.34			8-14 days	69 (17.9)	14.13±11.93		
Junior high school	128 (33.2)	11.54±9.86			15-21 days	23 (6.0)	15.96±12.84		
High school/Technical secondary school	93 (24.1)	15.05±10.37			More than 21 days	22 (5.7)	15.86±13.15		
College	49 (12.7)	20.16±11.44			Caregivers			2.892	0.022
Bachelor degree or above	26 (6.7)	27.27±11.63			Spouse	213 (55.2)	14.53±11.87		
Marital status			0.961	0.411	Child or son/daughter-in-law	122 (31.6)	10.42±10.22		
Married	343 (88.9)	13.08±11.58			Parents	34 (8.8)	15.47±11.74		
Spinsterhood	32 (8.3)	13.84±11.19			Other (Rotate care/hire carers)	17 (4.4)	12.53±12.70		
Divorce	5 (1.3)	21.40±14.42			Sources of information related to VTE			126.824#	0.000
Widowed	6 (1.6)	11.00±5.51			Doctors	84 (21.8)	19.37±12.30		
Occupation			12.280	0.000	Nurses	51 (13.2)	20.08±10.61		
Farmer	139 (36.0)	9.96±9.92			Family member/friend	40 (10.4)	17.70±10.83		
Worker	49 (12.7)	12.57±11.25			Internet/TV	47 (12.2)	11.36±7.55		
Self-employed	21 (5.4)	12.71±8.19			other patient	14 (3.6)	11.07±12.31		
Professional	34 (8.8)	24.26±12.32			Pamphlet/poster	18 (4.7)	17.28±9.73		
Administrator	16 (4.1)	25.94±11.48			Professional books	2 (0.5)	21.00±14.14		
Retiree	58 (15.0)	12.03±10.53			Never heard of VTE	130 (33.7)	5.41±7.37		
Other	69 (17.9)	13.01±11.15			Whether there is a doctor/nurse in the family			4.762	0.000
Family per capita monthly income (US dollars)			34.850#	0.000	Yes	35 (9.1)	21.83±11.92		
Less than $235	23 (6.0)	6.87±9.70			No	351 (90.9)	12.36±11.14		
$235 - $470	133 (34.5)	9.46±9.32			Personal history of VTE			6.475	0.000
$470 - $783	140 (36.3)	15.67±11.81			Yes	11 (2.8)	32.00±9.72		
More than $783	90 (23.3)	16.60±12.32			No	375 (97.2)	12.67±11.11		
Payment manner of the medical expenses			51.540#	0.000	Family history of VTE			2.077	0.039
Out-of-pocket medical	31 (8.0)	9.90±9.37			Yes	30 (7.8)	17.40±9.49		
Medical services at state expense	14 (3.6)	18.36±11.21			No	356 (92.2)	12.87±11.62		
Medical insurance for urban workers	74 (19.2)	21.51±12.37			Whether the health-care providers provided the VTE knowledge education			8.095	0.000
The medical insurance for urban residents	81 (21.0)	13.49±11.85			Yes	130 (33.7)	19.72±11.87		
New rural cooperative medical insurance	170 (44.0)	9.82±9.41			No	256 (66.3)	9.93±9.83		

Notions: #: *H-*value

#### Reliability and validity

**Fig. 1:**
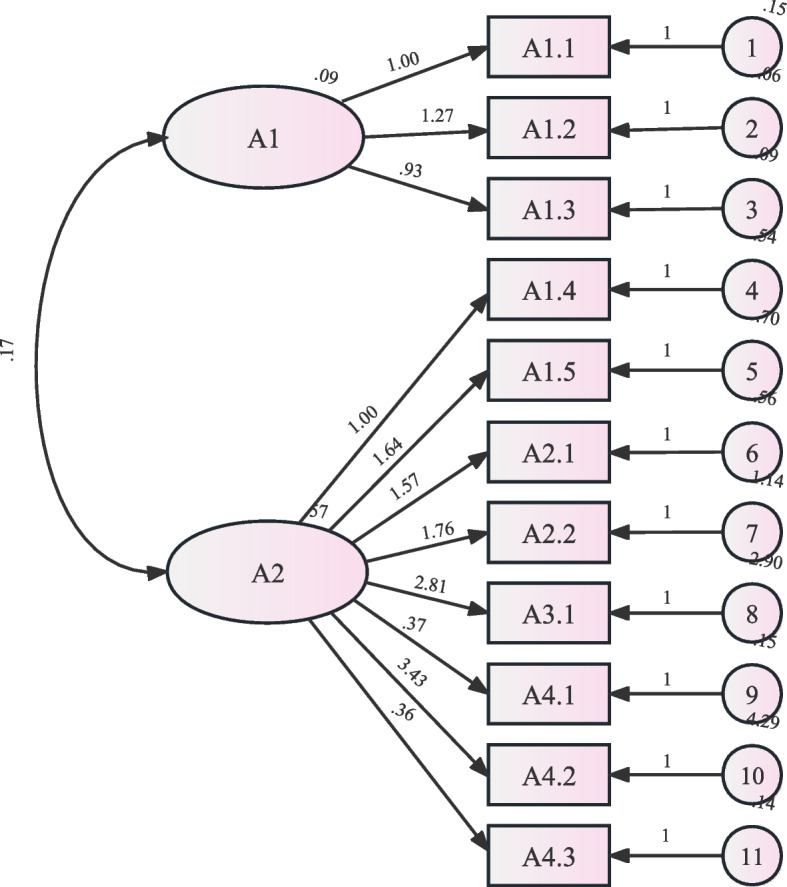
VTE knowledge questionnaire 2 factor confirmatory factor analysis model

The researchers invited 10 experts (2 clinical doctors, 3 nursing administrators, and 7 clinical nurses) to help to assess the content validity by means of a quantitative method in which the Content Validity Index (CVI) was used. Version 1 of the questionnaire’s item-level CVI is 0.40–1.00, scale-level CVI is 0.86. All comments and suggestions were considered to omit potential misunderstandings. After revision the version 2 of the questionnaire contains 11 questions, item-level CVI is 0.70–1.00, scale-level CVI is 0.95, demonstrating good content validity. Confirmatory factor analysis (CFA) was performed on 210 pilot sample cases, and fitting results showed that ^2^/df (^2^, goodness of fit test) was 3.318, IFI (Increasing Fitting Index) was 0.921, CFI (Comparative Fit Index) was 0.920, AGFI (Adjusted Goodness of Fit Index) was 0.821, all of them reached the ideal standard, indicating that the fitting degree and stability of the questionnaire fitting model are good (Fig. 1). The reliability of the final version questionnaire was evaluated using fractional reliability coefficients, Cronbach’s alpha coefficients, and retest reliability. A value of 0.70 or above of Cronbach’s alpha was considered evidence of internal consistency. Cronbach’s alpha coefficient of the questionnaire is 0.851, the fractional reliability coefficient is 0.885. Thirty patients were selected and measured again 7–10 days later. The results showed that the retest reliability is 0.791, indicating that the questionnaire has good stability.

#### Data analyses

All data analysis was conducted using Statistical Package for the Social Sciences (SPSS) version 26.0 and Analysis of Moment Structure (AMOS) version 27.0. For general data, frequency and percentage were used for descriptive analysis, mean, standard deviation and scoring rate was used for descriptive analysis of VTE knowledge score of neurosurgical hospitalized patients. (scoring rate = average score / total score × 100%, scoring rate < 60%, 60%-79%, > 80% corresponding low level, medium level, high level [17], respectively). The differences of VTE knowledge scores among hospitalized patients of neurosurgery in different age and gender, education level, economic status, and whether there was a doctor/nurse in the family, etc. were analyzed and compared using a t-test (t’ test was used when variance was not uniform) or Analysis of Variance (ANOVA), Kruskal–Wallis *H* test was used when variance was not uniform.

**Fig. 2:**
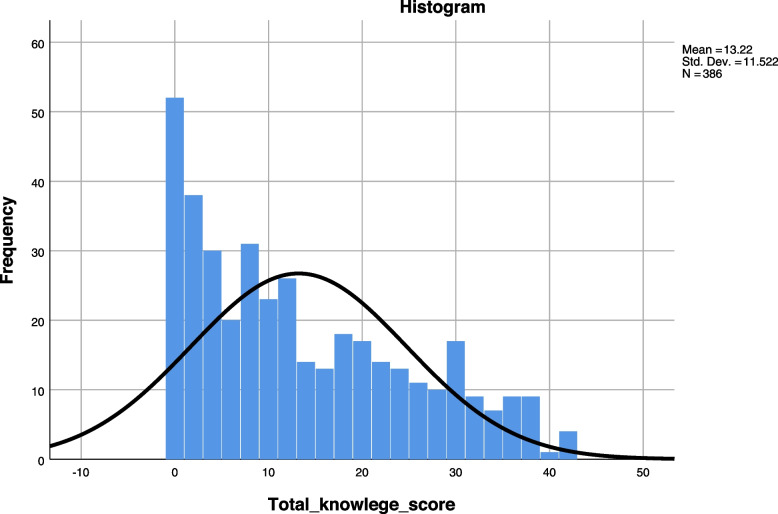
The histogram for the total knowledge score

The primary outcome variable for our research is a count of correct answers based on responses to a series of 11 questions assessing knowledge of VTE. Five of these items were counted as correct or not based on Yes or No responses to simple one-sentence questions. Six items consisted of a list of possible answers to general questions (e.g., Which of the following help prevent VTE?). A total of 41 questions were scored with 1 point for each correct answer giving a possible range of knowledge scores from 0 to 41. The Fig. 2 below showed the distribution of the counts of correct answers in our sample. The distribution type of the dependent variable was an example of an over-disbursed count distribution in which the variance (132.76) was substantially greater than the mean (13.22). Stepwise regression analysis and Negative Binomial Regression (NBR) were performed to analyze the factors influencing the knowledge level of VTE prevention in neurosurgical hospitalized patients. The process was divided into two steps: 1) In the first stage, all variables were taken as independent variables, and the natural logarithm of VTE prevention knowledge score was taken as dependent variable to conduct stepwise regression analysis; 2) At the second stage, the variables that were significant predictors in the first stage were the predictors in the NBR. Table 3 shows how the independent variables were coded. *P* values < 0.05 were considered as statistically significant in this study. Because we make multiple tests, the true significance level should be 0.05 divided by the maximum number of tests: 0.05/12 = 0.004.

**Table 3:** Independent variable coding table

Independent variable	Variable	Assignment instructions
Age (years)	X1	The original value
Place of residence	X2	Rural areas = 0, Urban areas = 1
Education Level	X3	Setting dummy variables (Use ‘Primary and below’ as controls)
Occupation	X4	Setting dummy variables (Use ‘other occupations’ as controls)
Family per capita monthly income (US dollars)	X5	Less than 1500 = 0, 1501–3000 = 1, 3001–5000 = 2, more than 5000 = 3
Payment manner of the medical expenses	X6	Setting dummy variables (Use ‘other manners’ as controls)
Surgery	X7	No = 0, Yes = 1
Caregivers	X8	Setting dummy variables (Use ‘other caregivers’ as controls)
Sources of information related to VTE	X9	Setting dummy variables (Use ‘never heard of VTE’ as controls)
Whether there is a doctor/nurse in the family	X10	No = 0, Yes = 1
Personal history of VTE	X11	No = 0, Yes = 1
Family history of VTE	X12	No = 0, Yes = 1
Whether the health-care providers provided the VTE knowledge education	X13	No = 0, Yes = 1

## Results

### Characteristics of participants

A total of 386 neurosurgical hospitalized patients completed the survey. The patient demographic and clinical information are shown in Table 2. The mean age of patient participants was 51.45 (SD = 13.65) years with a range of 19 to 86 years. Two hundred (51.8%) of these patients were female. One hundred and sixty-eight participants (43.5%) had a high school or college education. One hundred and ninety-five patients were postoperative (50.5%). The most common reason for admission was intracranial tumors (232, 60.1%). Most patients have no close family or friends with a history of VTE (356, 92.2%). Nearly two-thirds of patients (256, 66.3%) reported that they had not been informed about VTE by doctors/nurses. However, by asking nurses, it was found that when patients were admitted to the hospital, nurses would use a common VTE health education sheet (including basic knowledge of VTE and VTE preventive measures, etc.) to verbally inform patients to prevent VTE and the patient would sign it.

Twenty-seven (6.99%) patients were diagnosed with VTE by ultrasonography after admission. The medical chart review indicated that eleven patients had a previous VTE and two hundred and thirty-two patients had an intracranial tumor. Approximately 83.62% of the 232 patients who had an intracranial tumor did not select ‘cancer/chemotherapy/radiotherapy’ as a risk factor for VTE. In our study, all postoperative patients received mechanical prophylaxis including graduated compression stockings (GCS) and/or intermittent pneumatic compression (IPC) to prevent VTE, sixty-three patients (32.31%) of 195 postoperative patients chose ‘GCS or IPC’ as the preventive measure of VTE. Twenty-nine patients received pharmacological prophylaxis including direct oral anticoagulants (DOACs) \ injection of anticoagulant (low-molecular-weight heparin 2000 u/every time, one time/d, unfractionated heparin (UFH)/1.25 wμ, sixteen of them (55.17%) were unaware of the risk of bleeding from anticoagulants.

### Knowledge of VTE among neurosurgical hospitalized patients

Table 4 presents patient responses to the questions about VTE knowledge. Almost 53.6% of participants reported they had heard of VTE, almost 36.0% of participants reported they had heard of DVT, and nearly one quarter (21.2%) of participants reported that they had heard of PE, and 164 participants (42.5%) had never heard of either condition. Data about knowledge of the causes of VTE indicated that the most frequently identified reason for each group was ‘abnormal blood constituents’ (149, 38.6% of participants), followed by ‘abnormal blood flow’ (107, 27.7%), ‘abnormal blood constituents’ (66, 17.1%), and more than half of respondents (54.4%) reported that they had no idea why VTE happens. 44.3% of participants believed that VTE may be associated with complications, 38.1% of participants believed that PE is considered to be life-threatening.

**Table 4:** Awareness of VTE

Item	No.of responses	Percent	Item	No.of responses	Percent
A1.1 Have you heard of VTE?			A3.1 Which of the following might increase your risk of developing VTE?		
Yes	207	53.6	Immobility or bed rest for more than 3 days	193	50.0
No	179	46.4	Trauma	107	27.7
A1.2 Have you heard of DVT?			Surgery	159	41.2
Yes	139	36.0	Obesity	114	29.5
No	247	64.0	Use dehydrating drugs	42	10.9
A1.3 Have you heard of PE?			Deep vein catheterization	42	10.9
Yes	82	21.2	Have high blood pressure or diabetes and so on	106	27.5
No	304	78.8	Infection	54	14.0
A1.4 Which of following cause VTE?			Cancer/Chemotherapy/Radiotherapy	62	16.1
Abnormal blood flow	107	27.7	Personal history of VTE	105	27.2
Vessel wall abnormalities	66	17.1	Not sure	119	30.8
Abnormal blood constituents	149	38.6	A4.1 Taking VTE prevention measures will reduce the occurrence of VTE		
Not sure	210	54.4	Yes	242	62.7
A1.5 Which of following are the harm caused by VTE?			No	144	37.3
Affect physical recovery	182	47.2	A4.2 Which of following help prevent VTE?		
Prolonged hospital stays and increased medical costs	139	36.0	Drinking plenty of fluids (If the condition permits)	212	54.9
Complications such as post-thrombotic syndrome occurred	171	44.3	Quitting smoking and alcohol	184	47.7
Lead to the occurrence of PE which can be life-threatening	147	38.1	Pay attention to diet and control blood sugar and lipids	190	49.2
Not sure	140	36.3	Stretching and moving their legs during the period of stay in bed	172	44.6
A2.1 Which of following are signs/symptoms of DVT?			Raise the lower limb during the period of stay in bed	158	40.9
Swelling	155	40.2	Getting out of bed as early as possible	176	45.6
Calf pain	128	33.2	Using graduate compression stockings (GCS)	74	19.2
Redness and warmth of leg (s)	67	17.4	Using intermittent pneumatic compression (IPC)	84	21.8
Lower extremity varicose veins	142	36.8	Using direct oral anticoagulants (DOACs)	67	17.4
Not sure	160	41.5	Injection of anticoagulant	66	17.1
Difficulty breathing	147	38.1	A4.3 The use of anticoagulants to prevent VTE may have the risk of bleeding and other adverse effects		
Cough	80	20.7	Yes	91	23.6
Chest distress	113	29.3	No	295	76.4
Chest pain	111	28.8			
Coughing up blood	72	18.7			
Not sure	188	48.7			

Notions: Due to the limitation of the table width, the English abbreviation in this table is the full name in the actual questionnaire

The most frequently reported signs and symptoms of DVT was ‘swelling’ (40.2%), followed by ‘Lower extremity varicose veins’(36.8%), ‘calf pain’ (33.2%), and ‘redness and warmth of leg(s)’ (17.4%). About 36.7% of participants chose just one or two correct DVT signs and symptoms, 13.2% of them identified three correct answers, 8.5% of them identified all four correct answers, about 41.5% of them were not able to identify correctly any symptoms of DVT. When asked about signs and symptoms of PE, Participant responses to identifying signs and symptoms varied, with ‘difficulty breathing’ (38.1%) the most frequent, followed by ‘chest distress’ (29.3%), ‘chest pain’ (28.8%), ‘cough’ (20.7%), and ‘coughing up blood’ (18.7%) for patients. About 26.7% of participants identified at least one or two correct answers, 16.9% of them chose three or all four correct answers, 7.8% of them participants selected all five correct answers, about 48.7% of them were not able to identify correctly any symptoms of PE. Overall, approximately 38.9% of neurosurgical hospitalized patients were unable to correctly identify any symptoms of VTE.

Data about knowledge of the risk factors indicated that the most frequently identified risk factor was ‘immobility or bed rest for more than 3 days’ (50.0% of patients). Further risk factors identified were: ‘Surgery’ (41.2% of patients), and Obesity (29.5% of patients). The least frequently reported risk factor for VTE was ‘Use dehydrating drugs’ and ‘deep vein catheterization’ (10.9% of patients, respectively). Overall, approximately 30.8% of neurosurgical hospitalized patients were unable to correctly identify any risk factors of VTE.

When participants were asked about whether taking preventive measures for VTE will reduce the incidence of the disease, 62.7% of patients agreed with that. The majority of participants reported ‘drinking plenty of fluids (If the condition permits)’ would help prevent a VTE (54.9% of patients), followed by ‘paying attention to diet and control blood sugar and lipids (49.2%)’, ‘quitting smoking and alcohol’ (47.7%), ‘getting out of bed as early as possible’ (45.6%), ‘stretching and moving their legs during the period of stay in bed’ (44.6%), ‘raising the lower limb during the period of stay in bed’ (40.9%), the two that are least reported were ‘using direct oral anticoagulants (DOACs)’ (17.4%) and injection of anticoagulant (17.1%). The average score of basic preventive measures was 2.83 (SD = 0.12), followed by mechanical prophylaxis 0.41 (SD = 0.04), chemo-prophylaxis 0.34 (SD = 0.04), and the scoring rate were 47.17%, 20.50%, 17.00%, respectively. Overall, approximately 29.5% of neurosurgical hospitalized patients were unable to correctly identify any preventive measures of VTE. More than two-thirds of neurosurgical hospitalized patients (76.4%) were unaware of the risk of bleeding associated with taking anticoagulants to prevent VTE.

### Sources of information related to VTE

The most frequent source of information was doctors (84, 21.8%), followed by nurses (51, 13.2%), Internet/TV (47, 12.2%), family members/friends (40, 10.4%), pamphlet/poster (18, 4.7%), another patient (14, 3.6%), professional books (2, 0.5%). The ‘other’ choice was never heard anything about VTE.

### Knowledge level of VTE in neurosurgical hospitalized patients

The results showed that the knowledge level of VTE in neurosurgical hospitalized patients was at a low level, the average score was 13.22 (SD = 11.52), the scoring rate was 32.24%. The average score of four aspects (VTE disease concept, symptoms and signs, risk factors, prevention knowledge) were: 3.60 (SD = 0.17), 2.63 (SD = 0.15), 2.55 (SD = 0.14), 4.45 (SD = 0.20), respectively, and the scoring rate were: 36.0%, 29.2%, 25.5%, 37.1%, respectively.

### Analysis of influencing factors of VTE knowledge level in neurosurgical hospitalized patients

Table 2 displays the results of univariate analysis. The results showed that the ‘patient's age, place of residence, education level, occupation, family per capita monthly income, payment manner of the medical expenses, surgical, caregivers, sources of information related to VTE, whether there is a doctor/nurse in the family, personal history of VTE, family history of VTE, whether the healthcare providers provided the VTE knowledge education’ was significantly related to VTE knowledge level in neurosurgical hospitalized patients (*P* < 0.05).

Table 5 presents the results of a stepwise regression analysis. Results showed that 4 factors entered the stepwise regression equation: ‘education level, the sources of information related to VTE, Whether there is a health worker in patients’ family, personal history of VTE’, respectively. The model could explain 45.7% of the total variation. Model tolerance and variance inflation factor (VIF) were 0.581–0.938 and 1.066–1.722 respectively, indicating that there was no serious collinearity problem.

**Table5:** Stepwise regression analysis of influencing factors of VTE knowledge in neurosurgical hospitalized patients (*n* = 386)

Model	Unstandardized Coefficients		Standardized Coefficients	t	Sig	Correlations			Collinearity Statistics	
	B	Std. Error	Beta			Zero-order	Partial	Part	Tolerance	VIF
(Constant)	0.663	0.105		6.344	0.000					
Education level-College/Bachelor degree or above	1.296	0.153	0.414	8.466	0.000	0.330	0.401	0.322	0.606	1.651
Education level-High school/Technical secondary school	0.833	0.144	0.288	5.792	0.000	0.148	0.287	0.220	0.587	1.704
Education level-Junior high school	0.589	0.131	0.224	4.485	0.000	-0.034	0.226	0.171	0.581	1.722
VTE knowledge comes from doctors	1.012	0.136	0.337	7.440	0.000	0.259	0.359	0.283	0.706	1.417
VTE knowledge comes from nurses	1.339	0.155	0.366	8.657	0.000	0.245	0.408	0.329	0.810	1.234
VTE knowledge comes from family member/friend	1.172	0.170	0.288	6.902	0.000	0.144	0.336	0.263	0.830	1.204
VTE knowledge comes from internet/TV	0.659	0.160	0.174	4.117	0.000	0.024	0.208	0.157	0.810	1.234
VTE knowledge comes from other sources (including other patient/ Pamphlet and poster/ Professional books)	1.119	0.236	0.190	4.742	0.000	0.106	0.238	0.181	0.898	1.113
Personal history of VTE	0.875	0.292	0.118	2.990	0.003	0.185	0.153	0.114	0.938	1.066
Whether there is a doctor/nurse in the family	0.430	0.170	0.100	2.527	0.012	0.205	0.129	0.096	0.930	1.076

Notions: Dependent variable: Ln (Total VTE knowledge score), The model R^2^ = 0.457, *F* = 31.526, *P* = 0.000 < 0.001, input = 0.05, output = 0.10

Table 6 shows the results of the negative binomial regression. Combined with the results of parameter estimation, it can be seen that: 1) for education level, results showed that the higher the education level of patients, the higher the VTE knowledge was: junior middle school (*P* < 0.004, Odds Ratio(OR) = 1.627, 95% Confidence Interval (CI) = 1.207–2.193), high school/secondary [*P* < 0.001, OR = 2.108, 95%CI = 1.536–2.892), college diploma/bachelor [*P* < 0.001, OR = 3.095, 95%CI = 2.209–4.335], respectively; 2) The coefficient of “ VTE knowledge comes mainly from doctors” was -0.215 (*P* = 0.248 > 0.05), indicating that the influence of doctors' VTE education on VTE prevention knowledge of neurosurgery inpatients was not statistically significant; And patients who reported VTE knowledge comes mainly from ‘the nurse [*P* < 0.001, OR = 3.103, 95% CI = 2.201–4.374], family/friends [*P* < 0.001, OR = 2.971, 95% CI = 2.038–4.331], Internet/TV [*P* < 0.001, OR = 1.979, 95%CI = 1.382–2.834] and other approaches (including other patients/brochures and posters/professional books) [*P* < 0.001, OR = 2.236, 95%CI = 1.492–3.350]’ scored higher VTE knowledge score.

**Table 6:** Negative binomial regression of influencing factors of VTE knowledge in neurosurgical hospitalized patients (*n* = 386)

Parameter	*B*	*S.E*	95% Wald Confidence Interval		Hypothesis Test			OR	95% Wald Confidence Interval for OR	
			Lower	Upper	Wald Chi-Square	df	*P*		Lower	Upper
(Intercept)	1.122	0.1328	0.862	1.382	71.342	1	0.000	3.071	2.367	3.984
Whether there is a doctor/nurse in the family	0.411	0.1890	0.040	0.781	4.726	1	0.030	1.508	1.041	2.184
Personal history of VTE	0.861	0.3212	0.231	1.490	7.178	1	0.007	2.365	1.260	4.438
VTE knowledge comes from doctors	-0.215	0.1865	-0.581	0.150	1.333	1	0.248	0.806	0.559	1.162
VTE knowledge comes from nurses	1.132	0.1752	0.789	1.476	41.796	1	0.000	3.103	2.201	4.374
VTE knowledge comes from family member/friend	1.089	0.1923	0.712	1.466	32.046	1	0.000	2.971	2.038	4.331
VTE knowledge comes from internet/TV	0.683	0.1831	0.324	1.042	13.907	1	0.000	1.979	1.382	2.834
VTE knowledge comes from other sources (including other patient/ Pamphlet and poster/ Professional books)	0.805	0.2063	0.400	1.209	15.217	1	0.000	2.236	1.492	3.350
Education level-College/ Bachelor degree or above	1.130	0.1720	0.793	1.467	43.127	1	0.000	3.095	2.209	4.335
Education level-High school/ Technical secondary school	0.746	0.1614	0.429	1.062	21.335	1	0.000	2.108	1.536	2.892
Education level-Junior high school	0.487	0.1523	0.189	0.785	10.227	1	0.001	1.627	1.207	2.193
(Scale)	1^a^									
(Negative binomial)	1^a^									

^a^Fixed at the displayed value

## Discussion

Neurosurgical hospitalized patients have a significantly increased risk of VTE due to reduced preoperative activity, intraoperative immobilization, long-term postoperative bed rest, and extensive perioperative application of vascular stimulant drugs and dehydration drugs [20]. However, patients' understanding of VTE knowledge is not high. The results of this study showed that the average score of VTE knowledge of neurosurgical hospitalized patients was 13.22 (SD = 11.52), which was at a low level and needed to be further improved. Previous studies also investigated the knowledge level of VTE in orthopedic patients, cancer patients, and patients after cesarean section, and the results were not ideal. Our study is consistent with those studies [21–23].

The findings of our study indicate poor awareness of VTE, DVT, and PE among neurosurgical hospitalized patients (53.6%, 36.0%, and 21.2%, respectively). It's worth noting that nearly two-thirds of patients (256, 66.3%) reported that they had not been informed about VTE by doctors/nurses. However, by asking nurses, it was found that when patients were admitted to the hospital, nurses would educate them about VTE knowledge. This suggests that the efficiency of VTE education from health-care providers needs to be strengthened. Health-care providers need to focus on whether patients with actual to receive and understand their education of VTE knowledge. They need to consciously encourage patients to actively participate in the prevention process of VTE, cultivate awareness of patients' involvement in their safety management.

Our study also shows that more than half of respondents were unaware of the causes of VTE and that nearly two-thirds of the participants do not believe that blood clots can travel to the lungs and be life-threatening. This is consistent with the results of previous studies [24]. Correspondingly, they demonstrate the lack of awareness of the detailed information regarding its signs and symptoms: about 41.5% and 48.7% of neurosurgical hospitalized patients were not able to identify correctly any symptoms of DVT and PE, respectively. In terms of risk factors, most participants who correctly identified risk factors for VTE recognized ‘immobility or bed rest for more than three days’ as a key risk factor for DVT and PE development. The least frequently reported risk factor for VTE was ‘use dehydrating drugs’ and ‘deep vein catheterization’. This finding suggests the need to provide patients with more professional information on VTE to ensure a better understanding of its risks. In addition, about 60.1% (232) of the 386 neurosurgical hospitalized patients we investigated were patients with intracranial tumors, and approximately 83.62% of them did not select ‘cancer/chemotherapy/radiotherapy’ as a risk factor for VTE, indicating that the awareness of cancer as a risk factor for VTE was poor among intracranial tumor patients. Studies have shown that VTE occurs in up to 20% of patients with intracranial tumors every year, and the incidence of VTE is highest in patients with intracranial tumors compared with other tumor types [25, 26], thus, intracranial tumor patients should receive more VTE information. In terms of VTE prophylaxis, compared with mechanical prevention and drug prevention, basic preventive measures had better cognition, with a score of 47.17%. The most commonly reported preventive measures were ‘drinking plenty of fluids (If the condition permits)’. Among 195 postoperative patients who received VTE mechanical prophylaxis including graduate compression stockings (GCS) and/or intermittent pneumatic compression (IPC), only 63 patients (32.31%) chose ‘GCS or IPC’ as the preventive measure of VTE. This indicates that patients are less knowledgeable about the preventive measures for VTE, they only know to follow the doctors' advice but do not know why these measures are needed. Of 29 patients who were previously or currently receiving thrombotic drug prophylaxis, sixteen (55.17%) were unaware of the risk of bleeding associated with anticoagulants, which may reflect the failure of healthcare providers to provide patients with counseling regarding their treatment during hospitalization. In general, neurosurgical hospitalized patients have insufficient knowledge of VTE, especially in two aspects: ‘VTE signs and symptoms, risk factors’, with a scoring rate of 29.2% and 25.5%, respectively. The reason may be that nurses may pay more attention to the education of VTE prevention measures when conducting health education for patients, but do not popularize relevant clinical manifestations and risk factors. Therefore, it is necessary to strengthen the education of VTE knowledge in these aspects.

The negative binomial regression analysis results showed that among the patient demographic variables, only education level influences the neurosurgical hospitalized patient's level of knowledge of VTE. The reason may be that patients with higher education have a wider range of knowledge, a stronger thirst for knowledge and learning ability, and a stronger ability to obtain effective propaganda and education in communication with health-care providers. As neurosurgical hospitalized patients tend to be older and less educated, nurses/doctors should be encouraged to increase their efforts in health education, use easy-to-understand language, patiently explain many times, and combine with various forms of health education methods such as health education manual and propaganda course of small lectures. For patients with higher education levels or young patients, new health education methods such as public platforms and smartphone mobile medical applications can be used to improve their cognitive level of VTE knowledge. Our study also found that neurosurgical hospitalized patients’ VTE knowledge mainly came from doctors (21.8%). However, the results showed that VTE knowledge came from doctors did not affect patients' VTE knowledge scores, and the VTE knowledge score of such patients was lower than that of patients whose VTE knowledge was derived from nurses, followed by friends and relatives. This needs to be taken seriously. This suggests that doctors' efforts to carry out VTE health education are insufficient, it may be due to insufficient attention paid to communication between doctors and patients and busy clinical work. Previous studies have shown that clinicians' knowledge of VTE, especially the clinical manifestations of VTE and the identification of risk factors, is not ideal and at a low level [27]. Therefore, VTE prevention training of medical staff, especially doctors, should be strengthened to give full play to the guiding role of doctors and the linkage of medical care.

Our study has some limitations. First, there were no distractors or wrong answers and all the questions were closed-ended survey questions, so participants may have guessed correctly rather than answer base on their knowledge, even if they were not knowledgeable about the topic. To minimize guessing, the interviewers attempted to ask the survey questions in an open-ended manner, to allow enough time for the participants to think before providing their answers. Secondly, we covered only one tertiary level hospital’s neurosurgical hospitalized patients, it is difficult to know just how representative this sample is of all those patients undergoing neurosurgical hospitalized patients. In addition, some new admissions may be influenced by the presence of patients in the same ward who have already been investigated. Thus, studying more hospitals is recommended as a part of future studies on this issue. Third, the survey data came from self-reports, that are difficult to associate with objective evidence. Last but not least, In our study, only twenty-nine patients (7.5%) received pharmacological prophylaxis, because of the overall acceptance rate for drug prophylaxis was low, there may be some bias in the results of this study.

## Conclusion

In this study, the questionnaire on VTE knowledge of neurosurgical hospitalized patients was developed scientifically and reasonably with good reliability and validity, which can be used to investigate the knowledge of VTE among clinical neurosurgical hospitalized patients. The knowledge level of VTE of neurosurgical hospitalized patients was low and far from favorable levels, and the scores of each part from high to low were: VTE knowledge, the disease concept, symptoms and signs, risk factors. Education level is an important factor affecting the VTE knowledge level of neurosurgical hospitalized patients, healthcare providers should pay more attention to the VTE knowledge education of neurosurgical hospitalized patients, pay more attention to patients with low educational levels. In addition, It is necessary to pay attention to the prevention training of VTE for doctors and improve their attention to VTE. In clinical work, targeted measures should be taken to improve neurosurgical hospitalized patient' s knowledge level of VTE, which is of great significance to promote patient safety and improve nursing quality.

## Data Availability

The data that support the findings of this study are available from Xiangya Hospital, CSU, but restrictions apply to the availability of these data, which were used under license for the current study, and so are not publicly available. Data are however available from the authors upon reasonable request and with permission of Xiangya Hospital, CSU.

## Abbreviations

VTE: Venous thromboembolism
SD: Standard Deviation
CI: Confidence Interval
DVT: Deep Venous Thrombosis
PE: Pulmonary Embolism
LMWH: Low Molecular Weight Heparin
ASH: American Society of Hematology
DOACs: Direct Oral Anticoagulants
GCS: Graduate Compression Stockings
IPC: Intermittent Pneumatic Compression
CVI: Content Validity Index
UFH: Unfractionated Heparin
SPSS: Statistical Package for the Social Sciences
AMOS: Analysis of Moment Structure
ANOVA: Analysis of Variance
CFA: Confirmatory Factor Analysis
CSU: Central South University
OR: Odds Ratio
